# The Impact of Internal Migration on under-Five Mortality in 27 Sub-Saharan African Countries

**DOI:** 10.1371/journal.pone.0163179

**Published:** 2016-10-26

**Authors:** Abukari I. Issaka, Kingsley E. Agho, Andre M. N. Renzaho

**Affiliations:** 1 School of Social Sciences and Psychology, Western Sydney University, Penrith, New South Wales, Australia; 2 School of Science and Health, Western Sydney University, Penrith, New South Wales, Australia; National Institute of Health, ITALY

## Abstract

**Objective:**

The literature on the impact of internal migration on under-five mortality in sub-Saharan Africa has been limited. This study examined the impact of internal migration on under-five mortality rate in 27 sub-Saharan African countries.

**Design:**

The analysis used cross-sectional data from the most recent Demographic and Health Surveys of 27 sub-Saharan African countries. Information on the number of live births and the number of under-five deaths in the five years preceding the surveys in these countries was examined. Using variables from which migration data were generated, four migration statuses were computed, and the impact of each migration status on under-five mortality was analysed by using multivariate Cox proportional hazards regression models.

**Results:**

Of the 96333 live births, 7036 deaths were reported. In the unadjusted model, we found that, compared to urban non-migrant mothers, hazard of under-five mortality was 20% [HR: 1.20; 95% confidence interval (CI): (1.06–1.35)], 40% [HR: 1.40; 95% CI: (1.29–1.53)], and 43% [HR: 1.43; 95% CI: (1.30–1.58)] higher among urban-rural migrant, rural non-migrant, and rural-urban migrant mothers respectively. The likelihood of children dying did not change considerably when country and demographic variables were adjusted for. However, after controlling for health care service utilization factors, the results remained consistently significant for rurality. That is, mortality rates remained significantly higher among children of rural non-migrant [(HR: 1.20; 95% CI: (1.08–1.33), P-value (p) < 0.001] and rural-urban migrant [HR: 1.29; 95% CI: (1.15–1.45), p < 0.001] mothers than those of urban non-migrant mothers.

**Conclusion:**

Although under-five child mortality rate declined by 52% between 1990 and 2015 (from 179 to 86 per1000 live births) in sub-Saharan Africa, the continent still has the highest rate in the world. This finding highlights the need to consider providing education and health care services in rural areas, when implementing interventions meant to reduce under-five mortality rates among internal migrant mothers.

## Introduction

Population mobility has been known to be an integral part of development of individuals and nations [1]. This is because it makes it possible for goals and aspirations of individuals, families and households to be met. In addition, population mobility is important to the efficient and effective functioning of cities and regions, labour markets and communities [2]. There are different forms of population mobility, ranging from local diurnal mobility, like commuting, to permanent relocations which cross international borders. In between this continuum is the movement of people that occurs within the confines a country, which falls under the general rubric of internal migration.

The Push-pull theory of migration, first articulated by Ravenstein [3] considers internal migration beyond a pure economic argument, and stresses the need to pay attention to wider social, cultural and political aspects that not only affect peoples’ lives, but also inform their choices of whether or not to migrate. Push factors include internal migrants’ inability to return to rural areas after training or working in urban areas, and seek to maximise their likelihood of better quality of life in urban areas. Push factors include, among others, job insecurity in rural areas (e.g. lack of employment opportunities or promotion), poor working conditions, (e.g. poor working facilities), poverty (e.g. agricultural decline, lack of funding opportunities or low salaries) and political factors (e.g. insecurity, wars, or ethnic and religious persecution). In this sense, rural outmigration is a survival strategy utilized by poor rural dwellers. In contrast, pull factors represent the opposite of push factors. These include, among other factors, job security (e.g. availability of jobs in urban areas and increased promotion opportunities) and better working conditions and urban settings. However, reduced economic opportunities in major sub-Saharan African cities and the increase in urban poverty despite the implementation of Structural Adjustment Programs, has led to urban outmigration- the migration from towns to rural areas [4, 5].

The health effects of rural-urban migration in Africa have been documented [3–5], and this category of internal migration still accounts for the majority of internal migratory movements. However, the emerging evidence is that internal migration patterns and their health effects have varied across the sub-continent [6, 7]. Most studies examining the health effects of internal migration across sub-Saharan Africa have mainly focused on rural-urban migration, with inconsistent findings. Some researchers have found that rural-urban migrants tend to be healthier than their local urban counterparts [8, 9]. When examined by setting, a pattern showing lower child mortality in urban than rural areas predominate the literature, suggesting that survival chances of children in rural areas could be enhanced by urban migration [10–15].

Some studies have found that children of rural-urban migrants experience a much higher risk of mortality than children of urban non-migrants, even after the mother has lived in the city for several years, and controlling for numerous potential confounding factors [16–19]. Other studies have hypothesised that the rural-urban migrants have on average lower child mortality before they migrate than rural non-migrants, and after migration to urban areas, child mortality levels drop to equal or to become lower than that of urban non-migrants, after adjusting for socioeconomic correlates [15, 18]. A previous analysis of DHS data from 17 countries [20] highlights a three-level relationship that exists between rural-urban migration and infant/child mortality. According to this study, the prospects of survival of rural-urban migrants were higher than that of rural non-migrants, but lower than those of urban non-migrants apparently due to the prevailing “healthy migrant hypothesis”. That is, there is a potential migration selection bias whereby rural-urban migrants represent a selective group of migrants with better mental and physical health status to cope with the stress in the new environment. Therefore, they are healthier than the average sending rural areas as well as the receiving urban population. The effect of community-level factors and the migrant effect have yet to be tested in sub-Saharan Africa. A better way of looking at the above factors is to examine the effect of individual, household and community level-factors, and to conceptualise migration in such as a way that the multidimensional effects of internal migration on child mortality can be examined. Therefore, the aim of the study was to examine the impact of internal migration on under-five mortality in sub-Saharan African countries. Specifically, the study examined the pathways through which internal migration may act to influence under-five mortality. Given the conflicting findings about the health conditions of internal migrants discussed in the forgoing discussion, we sought to test the following hypotheses: 1) urban exposure through internal migration would be associated with lower under-five mortality rate and 2) urban exposure to under-five mortality rate would be influenced by individual and household level factors (e.g. socio-economic status) and community-level factors (e.g. health care service utilization). Findings of our study would be vital to governments, non-governmental organizations and other stakeholders in implementing and monitoring intervention strategies aimed at minimising infant and child deaths due to internal migration of mothers. In addition, the findings could guide policy makers in the proper allocation of resources to mitigate the child mortality differentials due to internal migration of mothers.

## Methods

### Data sources and methodology

Our analyses used data from the most recent Demographic and Health Surveys (DHS) of 27 sub-Saharan Africa countries. These DHS are nationally representative, population-based surveys which are conducted in about 70 countries since 1982. The aim of the surveys is to collect demographic data and population health status of people, including fertility, family planning, maternal and child health, and childhood mortality [21, 22]. Our analysis was restricted to the most recent singleton births of mothers with complete information on migration status, within the five years preceding each survey. This yielded a weighted total of 96637 children. The most recent births were used in the analyses because those births had detailed information about use of health services, and to limit the potential for differential recall of events from mothers who delivered at different durations prior to the survey of each countries [24].

### Ethical Considerations

This study was based on analysis of secondary data, with all participant identifiers removed. The survey was approved by the ICF International Institutional Review Board (IRB), and the Ethics Committee of the Opinion Research Corporation Macro International, Incorporated, (ORC Macro Inc., Calverton, USA). Permission to use the DHS data in this study was obtained from ORC Macro Inc.

### Study inclusion and exclusion criteria

The choice of countries included in this current study was constrained by countries whose DHS datasets were recent, and had information on mothers’ previous place of residence (urban or rural) and the duration of stay of the mother in the current place of residence. After a critical examination of the DHS datasets, we obtained 27 countries whose DHS data were recent (last seven years, between 2009 and 2016) and adequate. The countries were Benin, Burkina Faso, Burundi, Cameroon, Cote d'Ivoire, Comoros, Congo DR, Gabon, Gambia, Ghana, Guinea, Lesotho, Malawi, Mali, Mozambique, Namibia, Niger, Nigeria, Rwanda, Sao Tome & Principe, Senegal, Sierra Leone, Tanzania, Togo, Uganda, Zambia and Zimbabwe. We excluded countries whose most recent DHS datasets were earlier than 2009.

### Descriptive study variables

#### Outcome variable

The main outcome variable was under-5 mortality (death between birth and 59 months of age). Binary outcome variable was used in the analysis. Child death was considered a ‘yes’ (= 1) if death occurred during the specified age period and a ‘no’ (= 0) if the child was alive throughout the specified age period.

#### Main study factor

The main study factor was internal migration. For the purpose of this study, an internal migrant was defined as a person who relocated between any combination of rural and urban areas for more than one month in the 12 months preceding the surveys [25]. The literature has suggested various forms of internal migration, based on Baggio [23]’s classifications: rural-urban migration or rural outmigration-(movement from the countryside to the city), inter-rural (movement from one countryside to another), urban-rural or urban outmigration (movement from the city to the countryside) and interurban (movement from one city to another). However, urban non-migrant dwellers and rural non-migrant dwellers need to be considered when examining the effect of internal migration on child mortality.

#### Potential covariates

Our choice of potential covariates was based on previous literature on determinants of childhood mortality, particularly in sub-Saharan countries [24–30]. These covariates were adapted to the available data in the DHS of the countries surveyed; and were categorised into demographic, country, socio-economic and health care utilization variables. Potential demographic covariates were: (a) maternal age, consisting of *20–29 years* and *30–49 years*, with *<20 years* as reference category; (b) birth order, consisting of *2*^*nd*^ *- 4*^*th*^ and *5*^*th*^ *or higher*, with *1*^*st*^*-born* was reference category; (c) gender of the child, consisting of *female*, with *male* as reference category and (d) perceived size of the baby, consisting of *average* and *large*, with *small* as reference category. Country variables were: the 27 sub-Saharan African countries, with Senegal as reference category. Socio-economic covariates were: (a) maternal and partner levels of education, consisting of *primary education* and *secondary education or higher*, with *no schooling* as reference category, (b) maternal working status, consisting of *working*, with *not working* as reference category and (c) household wealth index, consisting of *rich* and *middle*, with *poor* as reference category. Health care utilization covariates were: (a) place of delivery of the baby, consisting of *health facility*, with *home* as reference category (b) mode of delivery of the baby, consisting of *caesarean*, with *non-caesarean* as reference category, (c) type of delivery assistance, consisting of *traditional birth attendants* and *untrained personnel*, with *health professional* as reference category; (d) number of antenatal care visits, consisting of *1–3 times* and *none*, with *4+ times* as reference category; (e) number of postnatal care visits, consisting of *3–41 days* and *none*, with *0–2 days* as reference category and (f) tetanus toxoid (TT) injections during pregnancy, consisting of *Yes*, with *No* as reference category. Wealth index measured the economic status of the households interviewed in the survey. Information about ownership of a range of household assets was used to construct the wealth index. A weight (factor score) was assigned to each asset, which was generated through principal component analysis [31]. The resulting asset scores were standardized in with respect to a standard normal distribution with a mean of zero and standard deviation of one. A score for each asset was assigned to each household. These scores were then summed up for each of the households. In the DHS datasets for each country, the household wealth index was categorised into five quintiles: poorest, poorer, middle, richer and richest. However, in our analysis, this index was re-categorised into three groups: the bottom 40% of households were referred to as poor households (*poor*), the next 40% as middle households (*middle*) and the top 20% as rich households (*rich*).

### Statistical Analysis

Mortality rates categorised by the four categories based on migration status were estimated using a method similar to that described by Rutstein and Rojas [32]. This was followed by preliminary analyses of the frequency tabulation and Cox proportional hazards regression was conducted to determine the unadjusted Hazards Ratio (HR) of under-five mortality for potential confounding variables. As part of the multivariable analyses, and to examine the impact of internal migration on under-five mortality, we followed a procedure similar to those employed by Antai and colleagues [13] and Stephenson and colleagues [18] as described in Table 1. In the first stage (Model 1), only the study factor was featured. The second stage (Model 2) included the study factor and country. The third stage (Model 3) included the study factor and demographic covariates. The fourth stage (Model 4) included the study factor and socio-economic covariates. The fifth stage (Model 5) included the study factor and health care utilization covariates. The final stage included the study factor and all potential covariates associated with under-five mortality. The objective of this modelling strategy was to allow a comparison of the influence of each of the different sets of covariates on the relationship between internal migration and under-five mortality.

The hazard ratios (HRs) and their 95% confidence intervals (CIs) obtained from the adjusted Cox proportional hazards models were used to measure the effect of internal migration on under-five mortality. STATA software version 13.0 (Stata Corporation, College Station, TX, USA) was used in all analyses. All tests were two-sided, and results with p-values <0.05 were considered to be statistically significant.

**Table 1:** Potential covariates used in the multivariate survival model.

Model 1	Model 2	Model 3	Model 4	Model 5
Gross effects	Country*	Demographic	Socio-economic	Healthcare utilization
Migration status	Migration status	Migration status	Migration status	Migration status
		Birth order	Mother's level of education	TT vaccination in pregnancy
		Perceived size of child	Mother's working status	Place of delivery
		Sex of child	Father's education	Mode of delivery
		Mother's marital status	Household wealth index	Antenatal care services
		Mother's age at birth of first child		Postnatal care services
		Mother age at the time of interview		Type of delivery assistance

*Senegal, Nigeria, Sierra Leone, Ghana, Benin, Burkina Faso, Cote d'Ivoire, Guinea, Mali, Niger, Gambia, Togo, Burundi, Cameroon, Comoros, Congo DR, Gabon, Lesotho, Malawi, Mozambique, Namibia, Rwanda, Sao Tome & Principe, Tanzania, Uganda, Zambia, Zimbabwe

### Results

Information on 7036 under-five deaths was obtained for our analyses, the deaths ranging from 132 cases in Senegal [U-5 mortality rate (U5MR) = 36 per 1000 live births; 95% confidence interval (CI): (24–46)] to 223 in Zimbabwe [U5MR = 68 per 1000 live births; 95% CI: (52–84)] (Table 2).

**Table 2:** Under-5 mortality rates with 95% confidence intervals (CI) in 27 sub-Saharan African countries.

Country	Year of DHS	Number of U-5 deaths in the 5 years preceding the survey	Number of live births	U-5 mortality rate per 1000 live births (95% CI)
Senegal	2012–13	132	3677	36 (24–46)
Nigeria	2013	1185	12922	92 (73–110)
Sierra Leone	2013	550	4478	123 (101–145)
Ghana	2014	108	2520	43 (30–60)
Benin	2011–12	128	2132	60 (45–75)
Burkina Faso	2010	340	4003	85 (67–103)
Cote d'Ivoire	2011–12	259	2651	98 (78–117)
Guinea	2012	192	2211	87 (69–105)
Mali	2013	140	1948	72 (55–88)
Niger	2012	420	5222	80 (63–98)
Gambia	2013	124	3356	37 (25–49)
Togo	2013–14	135	1885	72 (55–84)
Burundi	2010	246	3546	69 (53–86)
Cameroon	2011	525	6622	79 (62–97)
Comoros	2012	33	580	57 (42–72)
Congo DR	2013–14	285	3884	73 (57–90)
Gabon	2012	111	2208	50 (36–64)
Lesotho	2009	183	1795	102 (82–122)
Malawi	2010	581	7379	79 (61–96)
Mozambique	2011	159	2294	66 (53–86)
Namibia	2013	86	1767	49 (35–62)
Rwanda	2014–15	124	3316	37 (25–49)
Sao Tome & Principe	2009	30	504	60 (44–75)
Tanzania	2010	208	3089	67 (51–83)
Uganda	2011	257	4064	63 (48–79)
Zambia	2013–14	272	4998	54 (40–69)
Zimbabwe	2010–11	223	3282	68 (52–84)

U-5: Under-five.

Univariate analysis (Table 3) showed that the U5MR was significantly higher among rural-urban migrant mothers compared with urban non-migrant mothers [HR: 1.43, 95% CI: (1.30–1.58)], and was also significantly higher among children from rural areas compared with those from urban areas [HR: 1.33, 95% CI: (1.24–1.43)]. Sierra Leonean children had the highest risk of dying, compared with children from the other countries [HR: 3.58, 95% CI: (2.75–4.66)]. The U5MR was significantly higher among children whose mothers had no schooling, compared with those whose mothers had secondary education of higher [HR: 1.60, 95% CI: (1.47–1.73)], and children whose mothers did not have any TT vaccinations when they were pregnant had higher U5MR, compared with those whose mothers had at least one TT injection during their pregnancy [HR: 2.16, 95% CI: (1.96–2.37)].

**Table 3:** Characteristics and unadjusted hazards ratios (HR) (95% CI) of under-five mortality and migration status in 27 sub-Saharan African countries.

			Unadjusted	
	n	%	HR (95% CI)	P
**Migration status**				
Urban non-migration	20970	21.8	1.00	
Rural non-migration	47419	49.2	1.40 (1.29–1.53)	<0.001
Urban-rural migration	9623	10.0	1.20 (1.07–1.35)	0.002
Rural-urban migration	18322	19.0	1.43 (1.30–1.58)	<0.001
Type of residence				
Urban	30593	31.8	1.00	
Rural	65741	68.2	1.33 (1.24–1.43)	<0.001
Country				
Senegal	3677	3.8	1.00	
Nigeria	12922	13.4	2.65 (2.06–3.41)	<0.001
Sierra Leone	4478	4.6	3.58 (2.75–4.66)	<0.001
Ghana	2520	2.6	1.21 (0.88–1.65)	0.235
Benin	2132	2.2	1.69 (1.23–2.30)	0.001
Burkina Faso	4003	4.2	2.45 (1.88–3.20)	<0.001
Cote d'Ivoire	2651	2.8	2.86 (2.09–3.91)	<0.001
Guinea	2211	2.3	2.51 (1.89–3.35)	<0.001
Mali	1948	2.0	2.07 (1.53–2.80)	<0.001
Niger	5222	5.4	2.30 (1.76–3.01)	<0.001
Gambia	3356	3.5	1.06 (0.74–1.52)	0.731
Togo	1885	2.0	2.05 (1.53–2.75)	<0.001
Burundi	3546	3.7	2.01 (1.51–2.68)	<0.001
Cameroon	6622	6.9	2.27 (1.75–2.95)	<0.001
Comoros	580	0.6	1.70 (1.00–2.90)	0.051
Congo DR	3884	4.0	2.10 (1.58–2.78)	<0.001
Gabon	2208	2.3	1.45 (1.04–2.03)	0.028
Lesotho	1795	1.9	2.99 (2.24–4.00)	<0.001
Malawi	7379	7.7	2.25 (1.72–2.93)	<0.001
Mozambique	2294	2.4	2.00 (1.48–2.70)	<0.001
Namibia	1767	1.8	1.41 (0.99–1.99)	0.056
Rwanda	3316	3.4	1.08 (0.80–1.46)	0.597
Sao Tome & Principe	504	0.5	1.71 (1.05–2.77)	0.030
Tanzania	3089	3.2	1.91 (1.43–2.56)	<0.001
Uganda	4064	4.2	1.83 (1.38–2.41)	<0.001
Zambia	4998	5.2	1.52 (1.14–2.01)	0.004
Zimbabwe	3282	3.4	1.99 (1.50–2.62)	<0.001
Household Wealth Index				
Rich	19685	20.4	1.00	
Middle	38279	39.7	1.37 (1.24–1.50)	<0.001
Poor	38379	39.8	1.64 (1.49–1.80)	<0.001
Mother's age at birth of 1st child				
< 20	57700	59.9	1.00	
20–29	36938	38.3	1.06 (0.81–1.38)	0.692
30–49	1675	1.7	1.22 (0.94–1.59)	0.137
Mother's level of education				
Secondary or higher	34687	36.0	1.00	
Primary	34046	35.3	1.33 (1.23–1.44)	<0.001
No schooling	27585	28.6	1.60 (1.47–1.73)	<0.001
Mother's working status				
Working	27632	29.3	1.00	
Not working	66832	70.7	1.03 (0.96–1.10)	0.469
Mother's marital status				
Never married	86021	89.3	1.00	
Formerly married	10310	10.7	1.07 (0.99–1.18)	0.121
Father's level of education				
Secondary or higher	29863	33.6	1.00	
Primary	26504	29.8	1.18 (1.09–1.28)	<0.001
No schooling	32451	36.5	1.42 (1.31–1.53)	<0.001
Birth order				
1st	22406	23.2	1.00	
2nd-3rd	45603	47.3	1.28 (1.20–1.37)	<0.001
4th-6th	28325	29.4	1.20 (1.12–1.30)	<0.001
Sex of child				
Male	48610	50.5	1.00	
Female	47724	49.5	1.13 (1.07–1.20)	<0.001
Perceived size of the baby				
Large	14963	15.8	1.00	
Average	41325	43.7	1.11 (1.04–1.19)	0.002
Small	38313	40.5	1.60 (1.48–1.74)	<0.001
**Health services variable**				
Place of delivery of baby				
Health facility	53152	61.9	1.00	
Home	32741	38.1	1.38 (1.29–1.47)	<0.001
Mode of delivery of baby				
Non-caesarean	89889	94.9	1.00	
Caesarean	4841	5.1	1.06 (0.93–1.22)	0.389
Type of delivery assistance				
Health professional	56937	65.1	1.00	
Non-health personnel	30499	34.9	1.26 (1.17–1.35)	<0.001
Antenatal clinic visits				
4+	24209	25.1	1.00	
1–3	22291	23.1	1.14 (1.03–1.26)	0.009
None	49834	51.7	1.85 (1.71–2.00)	<0.001
Timing of postnatal check-up				
0–2 days	21012	22.3	1.00	
3–41 days	17239	18.2	1.11 (0.99–1.24)	0.071
None	58387	59.5	1.80 (1.64–1.97)	<0.001
TT injection during pregnancy				
1	16891	17.5	1.00	
2+	36854	38.3	1.13 (1.02–1.26)	0.019
None	42590	44.2	2.16 (1.96–2.37)	<0.001

HR: Hazard ratio. P: P-value.

As shown in Table 4, fitting only migration status into model 1 resulted in a higher hazard of dying among children of rural non-migrant [HR: 1.40, 95% CI: (1.28–1.53), P < 0.001], urban-rural migrant [HR: 1.20, 95% CI: (1.06–1.35), P = 0.003] and rural-urban migrant [HR: 1.43, 95% CI: (1.30–1.58), P < 0.001] mothers, compared to children of urban non-migrant mothers. There was no significant change in the hazard of U5M among children when we adjusted for country (Model 2). The hazard of U5M was significantly higher among Sierra Leonean children [HR: 3.43, 95% CI: (2.65–4.44), P < 0.001], compared to Senegalese children. Model 3 contained migration status and demographic variables, with no significant change in the hazard of death among children of the different migrant mothers. With the introduction of socio-economic characteristics along with migration status (Model 4), there was no change in the likelihood of death among children of urban-rural migrant mothers, compared to children of urban non-migrant mothers. There were, however, lowering in the likelihood of U5M among children of rural non-migrant and rural-urban migrant mothers, compared to children of urban non-migrant mothers, although these were not statistically significant. Model 5 contained characteristics associated with health care utilization and migration status, with the hazard of U5M remaining lower among children of rural non-migrant [HR: 1.20, 95% CI: (1.08–1.33), P = 0.001] and rural-urban migrant [HR: 1.29, 95% CI: (1.15–1.45), P < 0.001] mothers and higher among children of mothers who did not receive any tetanus toxoid injection during pregnancy [HR: 1.54, 95% CI: (1.41–1.70), P < 0.001], compared to children of mothers who did. The hazard of U5M was also higher among children whose mothers did not attend antenatal care clinics [HR: 1.21, 95% CI: (1.11–1.33), P < 0.001] compared to children whose mothers did.

**Table 4:** Adjusted HR (95% CI) of the association between under-five mortality and migration status in 27 sub-Saharan African countries.

	Model 1 (Migration status)	Model 2 (Country)	Model 3 (Demographic)	Model 4 (Socio-economic)	Model 5 (Health care)
	HR (95% CI)	HR (95% CI)	HR (95% CI)	HR (95% CI)	HR (95% CI)
**Migration status**					
Urban non-migration	1.00	1.00	1.00	1.00	1.00
Rural non-migration	1.40 (1.29–1.53)	1.37 (1.26–1.49)	1.39 (1.27–1.52)	1.08 (0.97–1.20)	1.20 (1.07–1.33)
Urban-rural migration	1.20 (1.06–1.35)	1.20 (1.07–1.35)	1.20 (1.06–1.36)	1.21 (1.06–1.37)	1.20 (1.05–1.38)
Rural-urban migration	1.43 (1.30–1.58)	1.36 (1.24–1.50)	1.41 (1.27–1.56)	1.10 (0.98–1.24)	1.29 (1.14–1.45)
**Community-level variable**					
*Country*					
Senegal		1.00			
Nigeria		2.62 (2.05–3.34)			
Sierra Leone		3.43 (2.65–4.44)			
Ghana		1.23 (0.91–1.66)			
Benin		1.69 (1.25–2.30)			
Burkina Faso		2.35 (1.81–3.05)			
Cote d'Ivoire		2.81 (2.07–3.82)			
Guinea		2.41 (1.82–3.18)			
Mali		1.96 (1.46–2.64)			
Niger		2.14 (1.65–2.79)			
Gambia		1.07 (0.75–1.54)			
Togo		2.03 (1.53–2.71)			
Burundi		1.86 (1.40–2.46)			
Cameroon		2.31 (1.79–2.98)			
Comoros		1.66 (0.98–2.82)			
Congo DR		2.00 (1.51–2.65)			
Gabon		1.60 (1.15–2.22)			
Lesotho		2.87 (2.16–3.81)			
Malawi		2.12 (1.64–2.75)			
Mozambique		1.95 (1.45–2.62)			
Namibia		1.44 (1.01–2.03)			
Rwanda		1.03 (0.77–1.38)			
Sao Tome & Principe		1.70 (1.05–2.75)			
Tanzania		1.85 (1.39–2.46)			
Uganda		1.71 (1.31–2.24)			
Zambia		1.49 (1.13–1.96)			
Zimbabwe		1.94 (1.45–2.55)			
**Household-level variable**					
*Household Wealth Index*					
Rich				1.00	
Middle				1.25 (1.12–1.39)	
Poor				1.43 (1.27–1.61)	
**Individual-level variable**					
*Mother's age (years)*					
15–24			1.00		
25–34			1.15 (1.05–1.26)		
35–49			1.07 (0.97–1.17)		
Mother's age at birth					
< 20			1.00		
20–29			1.08 (0.82–1.41)		
30–49			1.15 (0.87–1.52)		
*Mother's level of education*					
Secondary				1.00	
Primary				1.22 (1.10–1.35)	
No schooling				1.35 (1.21–1.52)	
*Mother's working status*					
Not working				1.00	
Working				1.03 (0.95–1.11)	
*Mother's marital status*					
Never married			1.00		
Formerly married			1.10 (1.00–1.21)		
*Father's level of education*					
Secondary				1.00	
Primary				0.98 (0.89–1.08)	
No schooling				1.09 (0.98–1.21)	
*Birth order*					
1st			1.00		
2nd-3rd			1.24 (1.14–2.35)		
4th-6th			1.11 (1.02–1.22)		
*Sex of child*					
Female			1.00		
Male			1.14 (1.07–1.21)		
*Perceived size of the baby*					
Large			1.00		
Average			1.11 (1.03–1.18)		
Small			1.58 (1.46–1.72)		
**Health services variable**					
*Place of delivery of baby*					
Health facility					1.00
Home					1.31 (1.12–1.52)
*Mode of delivery of baby*					
Non-caesarean					1.00
Caesarean					1.36 (1.17–1.58)
*Type of delivery assistance*					
Health professional					1.00
Non-health personnel					1.13 (0.97–1.31)
*Antenatal clinic visits*					
4+					1.00
1–3					1.03 (0.92–1.15)
None					1.20 (1.07–1.34)
*Timing of postnatal check-up*					
0–2 days					1.00
3–41 days					1.12 (0.99–1.27))
None					1.16 (1.03–1.30)
*TT injection during pregnancy*					
1					1.00
2+					1.20 (1.07–1.35)
None					1.74 (1.55–1.97)

Model 1: migration status; Model 2: migration status and countries; Model 3: migration status and demographic variables; Model 4: migration status and socio-economic variables; Model 5: migration status and health care utilization variables; Model 6: All potential covariates. HR: hazard ratio; CI: confidence interval. TT: tetanus toxoid.

## Discussion

Our study of large populations of mothers highlighted the impact of internal migration on U5MR in 27 sub-Saharan African countries. In general, hazards of under-five mortality was were higher among among urban-rural migrant, rural non-migrant, and rural-urban migrant mothers than urban non-migrant mothers. Our findings confirmed our first hypothesis that U5MR would be lower among mothers who lived in urban areas. Our second hypothesis that the effect of urban exposure on U5MR would be influenced by socio-economic factors was also confirmed. That is, our results remained significant after controlling for demographic factors and health care utilization. However, after controlling for socio-economic factors hazards for under five mortality for rural non-migration and rural-urban migration became non-significant but U5MR remained significantly higher among urban-rural migrant mothers than their urban non-migrant counterparts.

In our study, we found that U5MR was significantly higher among children of rural-urban migrant mothers who had no formal education, compared to those whose mothers had secondary education or higher. This is consistent with past research [33, 34], where child survival is a function of a higher level of maternal education. A past study estimated that of 8·2 million fewer deaths in children younger than 5 years between 1970 and 2009 in 175 developing countries, 4·2 million (51·2%) could be attributed to increased educational attainment in women of reproductive age [33].

We also found that when health care services utilization factors were adjusted for, the under-five mortality rates were significantly higher among children of rural-urban migrant mothers compared to children of urban non-migrant mothers. This is an indication of the fact that under-five mortality rates were driven by other factors other than internal migration. Further analysis revealed that the under-five mortality rates were significantly higher among children of rural-urban migrant mothers who did not have any TT vaccinations during pregnancy, compared with those whose mothers had one TT vaccination during pregnancy. Additionally, we found that the U5MR was significantly lower among children of rural-urban migrant mothers who were born at a health facility, compared to those who were born at home. This finding is consistent with findings from other previous studies [35, 36], and highlights the important role maternal immunization plays in preventing child deaths [37]. It is however, inconsistent with findings from a past study in Ethiopia, which revealed that children of rural-urban migrant mothers had lower immunization coverage compared to those from non-migrant mothers [38], and as immunization has been extensively reported in the extant literature to be a contributor to the reduction of childhood mortality [39], would have a higher mortality rate.

In our study, there were other factors which influenced the impact of internal migration on U5MR. We found that U5MR was significantly higher among male children of urban-rural migrant mothers, compared to their female counterparts. A similar finding was made in a previous study in Kenya [40], where females were found to have lower mortality risks compared to males. No biological reasons have been attributed to these differences, because of disagreements with other studies which found no difference [41, 42]. The differences, however, could be explained by the gender specific conditions of the area concerned. We found that U5MR was significantly higher among children of younger mothers, consistent with a past study in Kenya [40], which found children from older mothers to have better survival compared to those from younger ones. The differential could be explained by the fact that older mothers possess the needed experience to give their children the appropriate health care they need compared to the younger ones [40]. Other factors for which U5MR was significantly high included household poverty, low level of maternal education, non-utilization of health care services such as antenatal care services and TT vaccinations.

Our study had several strengths. First, it used the data source of pooled national survey data of 27 countries of Sub-Saharan Africa, which yielded a sample size of sufficient power to examine under-five mortality rates in subgroups of interest. Second, the same core questionnaires were used in all DHS, which were conducted with consistent survey quality-control methods. Third, the possibility of recall bias of mothers was minimised by restricting the study population to the most recent births of mothers, five years before each survey, thereby increasing the validity of our study [43]. Fourth, a variety of socio-demographic and health service characteristics that might potentially confound the relationship between our main study factor and outcome was taken into consideration. This adjustment also resulted in an improvement of the validity of the analyses of the primary study factor. Finally, the Cox proportional hazard model was used to tease out the covariates for under-5 mortality. Modelling infant and child mortality incorporates a censoring process that other statistical techniques such as regression are unable to address.

In our study, we found that socio-economic and health care services utilization factors played a vital role in the decreased hazard of U5M among children of internal migrant mothers in sub-Saharan Africa. Our study, however, had a number of limitations. First, the DHS programme does not collect migration history directly, therefore we used basic information (relating to the period of time spent by a respondent in their current place of residence and whether their previous place of residence was a rural or urban setting) to establish migration status. Any mother who indicated her previous residence as rural and her current residence as urban was classified as a rural-urban migrant. A mother who did not relocate in the past 12 months and resided in a rural area was classified as rural non-migrant; the one who did not relocate during that period and resided in an urban area was classified as an urban non-migrant. Second, since a primary sampling unit was used as a proxy for cluster or community, the administratively defined boundaries may have misclassified individuals into inappropriate communities or clusters, and this could have possibly introduced some bias [44]. Third, our study did not cover interurban or inter-rural migration, as we were interested in urban-rural differentials only. Fourth, our study was subject to bias, as the DHS of the countries under study covered different time periods. Finally, a cause-effect relationship could not be established in this study due to the cross-sectional nature of the data.

### Policy Implications and Conclusions

Our study has highlighted the fact that in sub-Saharan Africa, the influence of internal migration on U5MR is driven mostly by socio-economic and health care services factors. There are a number of implications for policy. These include: 1) In socio-economic terms, formal education played an important role in child survival among rural-urban migrant mothers; 2) In terms of health care, U5MR among children of rural-urban migrant mothers was significantly higher among those who delivered their babies at home, those who did not attend any antenatal care clinics and those who did not take any TT vaccinations during their pregnancy. Governments and other stakeholders in countries of this sub-region should consider interventions aimed at improving socio-economic factors such as maternal education, and making important health care services such as TT vaccinations for mothers during pregnancy easily accessible and affordable.

## Supporting Information

S1 File (ZIP)
