# Correction: The Impact of Internal Migration on under-Five Mortality in 27 Sub-Saharan African Countries

**DOI:** 10.1371/journal.pone.0171766

**Published:** 2017-02-02

**Authors:** Abukari I. Issaka, Kingsley E. Agho, Andre M. N. Renzaho

## Notice of Republication

This article was republished on January 24, 2017, to replace an earlier version that was published in error. Please download this article again to view the correct version.
